# Tuberculosis Lymphadenitis and Human Immunodeficiency Virus Co-infections among Lymphadenitis Patients in Northwest Ethiopia

**DOI:** 10.4314/ejhs.v31i3.23

**Published:** 2021-05

**Authors:** Yohannes Zenebe, Yesuf Adem, Begna Tulu, Daniel Mekonnen, Awoke Derbie, Zewdie Mekonnen, Fantahun Biadglegne

**Affiliations:** 1 Department of Medical Laboratory Sciences, College of Medicine and Health Sciences, Bahir Dar University, Bahir Dar, Ethiopia; 2 Biotechnology Research Institute, Bahir Dar University, Bahir Dar, Ethiopia; 3 Biochemistry Department, College of Medicine and Health Sciences, Bahir Dar University, Bahir Dar, Ethiopia; 4 Leipzig university, Institute of Clinical Immunology, Germany

**Keywords:** Tuberculosis, lymphadenitis, Human immunodeficiency virus, Ethiopia

## Abstract

**Background:**

Tuberculosis and human immunodeficiency virus (HIV) are among the major health problems in Ethiopia. This study assessed the proportion of tuberculosis lymphadenitis (TBLN), HIV infection and their co-infection among TBLN presumptive individuals at the selected hospitals in Northwest Ethiopia.

**Methods:**

Institution based cross sectional study was carried out. Data on demographic and clinical variables were collected with standardized questionnaire. Microbiological culture was done on specimen obtained by fine needle aspirates. The HIV status was determined by rapid anti-HIV antibody test. Data was entered and scrutinized using SPSS version 20 statistical packages. A stepwise logistic regression model was used. The result was considered as statistically significant at P<0. 05.

**Results:**

A total of 381 lymphadenitis patients were included in the study. The overall prevalence of TBLN and HIV were at 250(65.6%) and 9(2.4%), respectively and their co-infection was at 6(2.4%). Based on the cytological examination, 301(79.0%) of them were diagnosed as TBLN. The age group, (P=0.01) and residency, (P=0.01) were found significantly associated with TBLN. Similarly, unsafe sex was also statistically significant for HIV infection (P=0.007).

**Conclusion:**

Tuberculosis lymphadenitis is the leading cause of TB and lymphadenitis in the region. However, TBLN-HIV coinfection was promisingly low. High rate of discrepancy was noticed between cytological and culture results. Hence, the TBLN diagnostic criteria shall pursue revision.

## Introduction

Tuberculosis (TB) is the most prevailing communicable disease worldwide. Around 8.8 million people develop TB and 1.45 million people die of TB annually (1). An increased incidence of TB has been reported in Africa and Asia, where the highest prevalence of co-infection with HIV and *M. tuberculosis* crop-up (2, 3). The global burden of TB related morbidity and mortality is common particularly in developing countries. According to the World Health Organization (WHO) report, about 31% of TB cases were in Sub- Saharan Africa and 15% of these being among people living with human immunodeficiency virus (PLWHIV) (4). Sub- Saharan Africa, including Ethiopia, is the highest prevalent area in TB infection. The Ethiopian Ministry of Health (MoH) report showed that, Ethiopia ranks third in Africa and 8^th^ among the 22 highest TB burdened countries in the world. Moreover, the prevalence of all forms of TB is estimated at 261 per 100,000 population, leading to an annual mortality rate of 64 per 100,000 population (5). According to the hospital statistical data of Ethiopian Federal MoH in 2008, TB was the leading cause of morbidity, the third cause of hospital admission and the second cause of death in Ethiopia (6).

While pulmonary tuberculosis (PTB) is the most common presentation, extra pulmonary tuberculosis (EPTB), especially TBLN, is also an important clinical phenotype (7). It is one of the prevailing types of EPTB, which is involved in different sites of the body (8–10). From the sites of infection with TBLN, cervical lymph node is identified as the prominent sites involved (11–13).

Human immunodeficiency virus infection is also the common health problem in sub-Saharan Africa, including Ethiopia (14). People who are HIV positive and infected with TB develop EPTB much more frequently, about 50% of cases (8, 14). The disruption of our immune system minimizes the ability of the granuloma formation which leads to increased bacterial growth and dissemination. Based on the review from Pittsburgh, the increase in pathology associated with HIV/TB co-infection is caused by a functional disruption of the local immune response within the granuloma (14).

Although TBLN and HIV are the major problems in our country, the real burden of the disease in the study area is not well explored at regional level. The data from these findings will enable the stakeholders to take evidence based masseurs on TB and HIV prevention strategies. Therefore, the plan of this study was to assess the proportion of TBLN, HIV, and TBLN-HIV co-infections among lymphadenitis patients attending at selected hospitals in Amhara Regional State.

## Materials and Methods

**Study area**: The study was conducted in selected hospitals of Amhara Regional State (Felege-Hiwot Referral Hospital (FHRH), University of Gondar Hospital, Debre-Markos Hospital and Gamby Hospital). Amhara region is one of the nine ethnic divisions of Ethiopia bordered by the nation of Sudan to the west, regions of Tigray to the north, Afar to the east, Benishangul-Gumuz to the west & southwest and Oromia to the south. Its capital is Bahir Dar which is located about 565 km away from Addis Ababa near Ethiopia's largest inland body of water, Lake Tana. Based on the 2007 Central Statistical Agency report, the Amhara Region has a population size of about 17,221,976 of whom 50.2% were men and 49.8% women; urban inhabitants accounts 12.27% of the population. The region has an estimated area of 159,173.66 square kilometers, with estimated density of 108. 2 people per square kilometer (15).

**Study design, period and population:** An institutional based cross sectional study was conducted from October 2017 through February 2017. The study population comprised all patients who had lymphadenitis and attended at the selected hospitals during the study period. Accordingly, a total of 381 study participants were included in this study.

**Data and specimen collection:** Sociodemographic characteristics and clinical variables were collected using a structured and predesigned questionnaire. The FNA sample was collected from the swollen superficial lymph nodes by using a 22-gauge needle with an attached 10-ml syringe. From each study participant, a small amount of FNA sample (approximately 5060 µl) was collected. Some portion of the specimen was used for the preparation of cytology smears on the site. The rest of the specimen was transferred into Nunc-Cryo tubes containing one ml of phosphate buffer saline (pH = 7. 4) for culture at Amhara Public Health Institute (APHI), Bahir Dar.

**Human Immunodeficiency Virus test:** Rapid immune-chromatographic based test was used to screen the HIV status of patients based on manufacturer's instructions. Based on the national algorithm of rapid test for Ethiopia, KHB was used as screening and positive samples were re-tested with STAT-PACK (Chembio HIV 1/2 STAT-PAK™ Assay, CHEMBIO DIAGNOSTIC SYSTEMS, INC., MEDFORD, NY, USA). Samples giving discordant results in the two tests were also reexamined using tie-breaker, (Uni-Gold HIV, Trinity Biotech PLC, Co. Wicklow, Ireland).

**Cyto-morphological staining and examination:** The FNA samples were smeared on clean slides on the spot of sample collection. Hematoxylin and eosin staining was performed based on the standard procedure. To evaluate whether the morphology was suggestive for tuberculous, the slides were examined by experienced pathologist. Cytological examination of FNA smears were considered diagnostic of TBLN when they contained a thick, yellowish material showing either necrotic background associated with the presence of lymphohistiocytic and the presence of a significant polymorphonuclear cell population. Moreover, the presence of a granulomatous inflammatory reaction consisting of giant cell, and/or epithelioid cell clusters and lymphohistiocytic cell population were considered (16).

**Mycobacterium culture:** The mixture of 1ml of FNA and phosphate buffer saline (pH = 7. 4) was transferred into falcon tube of 15 ml capacity. The samples were decontaminated from non-mycobacterial organisms by sodium dodecyl sulfate (17). The decontaminated samples were centrifuged at 3,000 rpm for 15 minutes. An aliquot of 100 µl of the neutralized samples were cultured into two LJ tubes (one with 0.6% pyruvate and one with 0.75% glycerol) for primary isolation of the organisms. The inoculated tubes were incubated at 35 to 37°C for 3 to 8 weeks and observed once a week for the growth of mycobacterium. Growth of the mycobacteria was confirmed by visual detection of colonial morphology and by microscopic examination of the colonies for acid fast bacilli (AFB) and cord formation. The growth was taken as mycobacterium tuberculosis (MTB) of white rough colonies with AFB and cord formation (17).

**Quality control:** The questionnaire was pretested before the actual study began. The collected data was checked daily for consistency and accuracy. For culture examination aseptically and strict follow up of each procedure was guaranteed and positive and negative controls were incorporated for comparison. To check for the quality of the LJ media, un-inoculated LJ tubes were incubated at the same time to control possible contamination. Cultures were considered as negative when no colonies have been seen after 8 weeks of incubation.

**Data processing and analysis:** Data entry and analysis was done using SPSS version 20 statistical packages. Descriptive statistics was used to determine the rate of TB lymphadenitis, HIV and TBLN-HIV co-infection. Bivariate analysis using binary logistic regression was carried out to determine the presence of a statistically significant association between explanatory variables and the outcome variables. Moreover, multivariate logistic regression model was executed to identify independently associated factors. All explanatory variables which were associated with the outcome variable in the bivariate analysis (P<0. 2) was included in the multivariate logistic regression model. Odds Ratio (OR), p-value and their 95% Confidence Intervals (CI) were calculated and the results were considered statistically significant at P<0.05.

**Ethics approval and consent to participate**: The protocol of the study was approved by the ethical review committee of Biotechnology Research Institute of Bahir Dar University with the reference number of ጤ/ሞ/ቴሸ/1/153/07 and letter of support was obtained from Amhara Regional Health Bureau. Written and informed consent/assent was obtained from each study participants/their guardian for both data collection and publication. The information provided by each respondent was kept confidential. Prior to HIV test, guidance and counseling was given for each participant by professionals. Positive results for microscopic and culture results were sent for diagnosis and prompt initiation of anti-TB drugs.

## Results

**Socio-demographic characteristics:** A total of 381 lymphadenitis patients were involved in this study. Among these, 243 (63.8%) of them were females. The mean age of the study population was 32.38±15.35 years (range from 4–84 years). Participants in the age range of 20–39 years were at 189(49.6%). Majority, 279(73.2%) of the study participants were people living in rural areas (Table 1).

**Table 1:** Socio-demographic characteristics of the study participants among lymphadenitis patients in Amhara National Regional State, Ethiopia

Variables	Frequency, N (%)
**Sex**	
Female	243(63.8)
Male	138(36.2)
**Age**	
<15	44(11.5)
15-19	35(9.2)
20-39	189(49.6)
>40	113(29.7)
**Residence**	
Urban	102(26.8)
Rural	279(73.2)
**Educational Level**	
Pre-school age	15(3.9)
Not read and write	250(65.6)
Primary school	71(18.6)
Secondary school	21(5.5)
Diploma and above	24(6.3)
**Marital status**	
Not married	99(26.0)
Married	246(64.6)
Divorced	24(6.3)
Widowed	12(3.1)
**Occupation**	
Merchant	28(7.4)
Student	53(13.9)
House wife	55(14.4)
Daily laborer	15(3.9)
Employee	11 (3.0)
Farmer	196 (51.4)
Others	23 (6.0)

**Figure 1:**
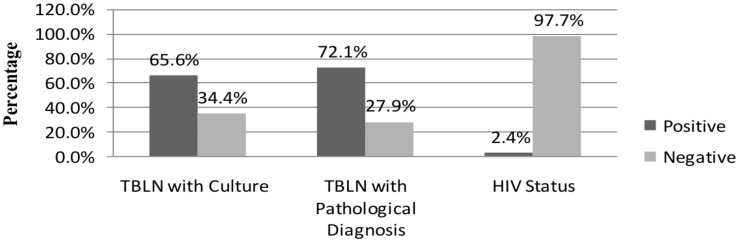
The proportion of TBLN and HIV among lymphadenitis patients in Amhara National Regional State, Northwest Ethiopia, 2017; TBLN: Tuberculosis Lymphadenitis, HIV: Human Immunodeficiency Virus

**Proportion of TBLN, HIV and TBLN-HIV co-infection:** The overall proportion of culture positive TBLN and HIV was 250(65.6%) and 9(2.4%), respectively (Figure 1). Among HIV positive patients, 6(66.7%) of them were also TBLN positive. However, the overall TBLN-HIV co-infection was 6/250(2.4%). With cytological examination, 301(79%) of the suspected patients were suggested to be TBLN. Tuberculosis lymphadenitis among the age groups of <15 years and > 40 years were, 35(79.5%) and 82(72.6%), respectively (Table 2).

**Table 2:** Prevalence of tuberculosis and HIV among lymphadenitis patients in Amhara National Regional State, Ethiopia

Variables	TB in culture		HIV status	
	Positive	Negative	Positive	Negative
**Sex**				
Female	154(63.4)	89(36.6)	7(2.9)	236(97.1)
Male	96(69.6)	42(30.4)	2(1.4)	136(98.6)
**Age**				
<15	35(79.5)	9(20.5)	1(2.3)	43(97.7)
15–19	23(65.7)	12(34.3)	0(0)	35(100.0)
20–39	110(58.2)	79(41.8)	5(2.6)	184(97.4)
>40	82(72.6)	31(27.4)	3(2.7)	110(97.0)
**Residence**				
Urban	59(57.8)	43(42.2)	2(2.0)	100(98.0)
Rural	191(68.5)	88(31.5)	7(2.5)	272(97.5)
**Occupation**				
Merchant	17(60.7)	11(39.3)	0(0)	28(100.0)
Student	37(69.8)	16(30.2)	1(1.9)	52(98.1)
House wife	32(58.2)	23(41.8)	2(3.6)	53(96.4)
Daily laborer	13(86.7)	2(13.3)	3(20.0)	12(80.0)
Employee	6(54.5)	5(45.5)	0(0)	11(100.0)
Farmer	122(62.2)	74(37.8)	2(1.0)	194(99.0)
Others	23(100.0)	0(0)	1(4.3)	22(95.7)
**Educational status**				
Pre-school age	13(86.7)	2(13.3	0(0)	15(100.0)
Not read and write	162(64.8)	88(35.2)	7(2.8)	243(97.2)
Primary school	44(62.0)	27(38.0)	1(1.4)	70(98.6)
Secondary school	15(71.4)	6(28.6)	0(0)	21(100.0)

The proportion of HIV among female and male patients was 7(2.9%) and 2(1.4), respectively. The proportion of HIV was also highest among patients came from rural areas, 7(2.5%) and daily laborers, 3(20%) (Table 2).

**Associated risk factors for TBLN and HIV infection:** Multivariate logistic regression analysis was conducted to assess independent risk factors for TBLN and/or HIV infections. But most of the variables have not statistically significant association. However, the age group, (AOR= 2.00, 95% CI, 1.17–3.43) and residency, (AOR=2.37, 95% CI, 1.20–4.65) were significantly associated with TBLN infection (Table 3). Regarding to HIV, unsafe sex was also statistically significant for HIV infection (AOR=7.25, 95%CI, 1.70–30.79) (Table 4).

**Table 3:** Associated factors of TBLN among tuberculosis lymphadenitis patients in Amhara National Regional State, Northwest Ethiopia

Variables	Total No.	Culture positive TB	COR(CI),p value	AOR(CI), p value
**Sex**				
Female	243	154(63.4)	1.32(0.84–2.06),0.22	1.25(0.70–2.22),0.44
Male	138	96(69.6)	1	1
**Age**				
<15	44	35(79.5)	0.68(0.29–1.58),0.37	0.68(0.14–3.24),0.63
15–19	35	23(65.7)	1.4(0.61–3.10),0.44	1.70(0.43–6.62),0.44
20–39	189	110(58.2)	1.9(1.14–3.14),0.01	2.00(1.17–3.43),0.01
>40	113	82(72.6)	1	1
**Residence**				
Urban	102	59(57.8)	1.58(0.99–2.52),0.05	2.37(1.20–4.65),0.01
Rural	279	191(68.5)	1	1
**Occupation**				
Merchant	28	17(60.7)	0.66(0.25–1.74),0.41	-
Student	53	37(69.8)	1.10(0.43–2.8),0.82	-
House wife	55	32(58.2)	0.23(0.04–1.28),0.09	-
Daily laborer	15	13(86.7)	1.28(0.31–5.26),0.72	-
Employee	11	6(54.5)	0.93(0.41–2.11),0.87	-
Farmer	196	122(62.2)	1	
Others	23	23(100.0)	-----	
**Educational status**				
Pre-school age	15	13(86.7)	0.30(0.06–1.7),0.17	2.61(0.14–48.88),0.52
Not read and write	250	162(64.8)	1.1(0.45–2.64),0.85	1.19(0.25–5.58),0.82
Primary school	71	44(62.0)	1.22(0.46–3.25),0.68	5.25(1.07–25.72),0.04
Secondary school	21	15(71.4)	0.8(0.22–2.85),0.73	0.80(0.16–3.85),0.78
Diploma and above	24	16(66.7)	1	1
**Marital status**				
Not married	99	72(72.7)	1.8(0.38–9.11),0.43	2.45(0.39–15.26),0.33
Married	246	151(61.4)	3.14(0.65–14.6),0.14	3.01(0.62–14.48),0.17
Divorced	24	17(70.8)	2.0(0.35–11.9),0.42	1.70(0.27–10,78),0.57
Widowed	12	10(83.3)	1	1
**Family size**				
≤4	164	110(67.4)	0.89(0.58–1.37),0.60	0.81(0.49–1.36),0.43
≤5	217	140(64.5)	1	1
**History of TB contact**				
Yes	122	80(65.6)	1.0(0.63–1.57),0.99	1.23(0.73–2.08),0.42
No	259	170(65.6	1	1
**History of TB**				
Yes	37	26(70.3)	0.79(0.37–1.65),0.53	0.63(0.27–1.45),0.27
No	343	223(64.9)	1	1
**History of raw milk**				
Yes	27	17(63.0)	0.88(0.53–1.45),0.62	0.96(0.53–1.74),0.91
No	354	233(65.8)	1	

**Key**: **COR**: Crude Odds Ratio, **AOR**: Adjusted Odds Ratio, **CI**: Confidence Interval, **TBLN**: Tuberculosis Lymphadenitis.

**Table 4:** Associated factors of HIV among tuberculosis lymphadenitis patients in Amhara National Regional State, Northwest Ethiopia

Variables	Total No.	HIV positives	COR(CI),p value	AOR(CI),p value
Sex				
Female	243	7(2.9)	0.49(0.10–2.42),0.38	0.36(0.07–1.89),0.23
Male	138	2(1.4)	1	1
Age				
<15	44	1(2.3)	1.17(0.11–11.58),0.89	0.47(0.02–10.69),0.63
15–19	35	0(0)	0.58(0.23–3.44),0.84	0.77(0.02–26.45),0.88
20–39	189	5(2.6)	1.00(0.23–4.28),0.99	3.10(0.47–20.40),0.23
>40	113	3(2.7)	1	1
Residence				
Urban	102	2(2.0)	1.28(0.26–6.29),0.75	1.03(0.17–6.20),0.97
Rural	279	7(2.5)	1	1
Occupation				
Merchant	28	0(0)	0.47(0.13–1.45),0.99	-
Student	53	1(1.9)	2.36(0.14–39.50),0.54	-
House wife	55	2(3.6)	1.20(0.10–13.97),0.88	-
Daily laborer	15	3(20.0)	3.24(0.35–12.65),0.97	-
Employee	11	0(0)	2.02(0.22–10.54),0.89	-
Farmer	196	2(1.0)	1.73(0.19–15.54),0.62	-
Others	23	1(4.3)	1	
Educational status				
Pre-school age	15	0(0)	0.98(0.44–15.34),0.95	074(0.54–14.36),0.85
Not read and write	250	7(2.8)	1.50(0.17–12.80),0.70	2.30(0.24–13.85),0.75
Primary school	71	1(1.4)	3.04(0.18–50.63),0.43	2.75(0.23–55.45),0.45
Secondary school	21	0(0)	2.40(0.29–16.24),0.94	2.40(0.29–16.25),0.94
Diploma and above	24	1(4.2)	1	1
Marital status				
Not married	99	2(2.0)	0.99(0.19–5.20),0.99	1.42(0.49–40.37),0.12
Married	246	5(2.0)	0.22(0.03–1.69),0.14	1.98(0.04–9.55),0.72
Divorced	24	2(8.3)	0.54(0.56–6.34),0.95	0.54(0.57–7.45),0.98
Widowed	12	0(0)	1	1
Family size				
≤4	164	4(2.4)	0.94(0.24–3.57),0.93	0.75(0.13–4.42),0.75
≤5	217	5(2.3)	1	1
Number of sexual partners				
1	121	4(2.1)	0.44(0.11–1.83),0.26	0.43(0.09–1.89),0.26
2	88	4(4.5)	0.98(0.10–9.01),0.99	2.07(0.19–21.81),0.54
≥3	47	1(2.1)	0.99(0.25–12.54),0.98	0.97(0.24–12.56),0.94
Not at all	55	0(0)	1	1
Unsafe sex*				
Yes	27	3(11.1)	7.27(1.70–30.79),0.007	7.25(1.70–30.79),0.007
No	354	6(1.7)	1	1

**Key**: **COR**: Crude Odds Ratio, **AOR**: Adjusted Odds Ratio, **CI**: Confidence Interval

* any sex without condom to different sexual partners

## Discussion

Tuberculosis and AIDS are the two most common infectious diseases throughout the world, especially in developing countries like Ethiopia. Tuberculosis lymphadenitis is increasing from time to time and its burden is exacerbated by HIV comorbidity. In the current study, the overall proportion of TBLN is still high, 65.6%. Reports showed that around 40% of EPTB cases are attributable to TBLN (18). This finding is much higher than the study conducted in Mozambique, 44.5% (19), Bangladesh, 52.3% (20) and Turkey, 56.3% (21). However it is comparable to the study conducted from the southern part of Ethiopia, 68.6% (22). The outcome of this study showed higher proportion of males (69.6%) affected with TBLN. This is consistent with the related published data in Ethiopia (23, 24) and other countries like India, 65.6% (25) and Nigeria, 64.6% (26). Even though, the reason for enhanced prevalence among males is controversial and uncertain, the differences in their social interaction and health seeking behavior may be considered as possible reasons.

According to the cytological examination, 301(79%) of the suspected patients were suggested to be TBLN and only 217(60.0%) of them were positive in both cytological and the TB culture diagnostic techniques. This shows that confirmatory tests of TBLN have to be incorporated to assure the appropriate diagnosis and treatment of patients.

The highest proportion of TBLN was identified among the age group less than 15 years, 35(79.5%) followed by the age group greater than 40 years, 82(72.6%) (Table 2). This might be related to the immune status of patients. Likewise, the highest proportion of HIV was identified at the age group greater than 40 years, 2.7%. Although marital status was not significantly associated, the uppermost prevalence of TBLN cases was observed among widowed individuals, 10(83.3%) and daily laborers, 13(86.7%). These groups of people are the most exposed group for TB and HIV infections. Hence, targeted intervention is recommended to such groups of population.

In this study, the overall prevalence of HIV among the study group was 9(2.4%) and the highest proportion was detected on females, 7(2.9%). This finding is higher than the national prevalence of HIV in Ethiopia, 1.1%, though there is a high regional variation (0.7%–6.4%) (27). However, it is less than the study done at Butajira Hospital, Southern Ethiopia, 6.3% (22). Even though, statistical significant association was not noted, the HIV proportion was also highest among patients came from rural areas and daily laborers. This result is an alarming circumstance which indicates the shift and increasing trend of HIV to rural areas. Most of the people from rural areas in the region are usually using a shared sharpen materials and have low awareness on the transmission of HIV (27). According to the Ethiopian Demographic and Health Survey of 2016, rural people are less likely than urban people to have knowledge about HIV transmission and prevention (28). In addition, daily laborers were also the most vulnerable and exposed group for sexually transmitted infectious diseases. Hence, priority supposed to be given for these groups of people for confronting the spread of HIV.

Studies have shown the correlation between HIV infection and TBLN (29–31). The synergies between HIV and TB infection have resulted in an increase in the incidence of TBLN and led for further complication on TB control strategies. Of the total HIV screened culture positive TB patients, the proportion of TB-HIV co-infection was 6 (2.4%). This finding was much less than other studies in Ethiopia (32, 33). The low prevalence might suggest a declining trend of HIV infection associated with TBLN in Amhara region.

In the current study, most of the sociodemographic variables were not significantly associated with TBLN infection. This might be due to the small sample size. However, the age group, (P=0.01) and residency, (P=0.01) were significantly associated with TBLN infection (Table 3). Similarly, unsafe sex was statistically significant for HIV infection (P=0.007) (Table 4).

In conclusion, TBLN remains a significant fraction of the total and extra pulmonary tuberculosis cases in Ethiopia. Moreover, the overall prevalence of HIV is relatively higher than the national prevalence (2.4% vs 1.1%). Fortunately, low prevalence of TBLN-HIV co-infection has been recorded.

The drug resistant pattern of isolates was not assessed. Moreover, the study fails to do molecular characterization and confirmation of *M. Tuberculosis* isolates to the species and lineage level. Hence, further study with large sample size and molecular diagnostic techniques is recommended.
